# Diversity and Distribution of Whiteflies Colonizing Cassava in Eastern Democratic Republic of Congo

**DOI:** 10.3390/insects13090849

**Published:** 2022-09-19

**Authors:** Clérisse M. Casinga, Everlyne N. Wosula, Mouritala Sikirou, Rudolph R. Shirima, Carine M. Munyerenkana, Leon N. Nabahungu, Benoit K. Bashizi, Henry Ugentho, Godefroid Monde, James P. Legg

**Affiliations:** 1 International Institute of Tropical Agriculture, Bukavu-Kalambo, Bukavu, Democratic Republic of the Congo; 2 Department of Environmental Sciences, Université du Cinquantenaire de Lwiro, Kabare, Bukavu, Democratic Republic of the Congo; 3 International Institute of Tropical Agriculture, Dar es Salaam, Tanzania; 4 International Institute of Tropical Agriculture, Kinshasa, Democratic Republic of the Congo; 5 Programme National Manioc, Institut National d’Etude et de Recherche Agronomiques de Mulungu, Bukavu-Mulungu, Democratic Republic of the Congo; 6 Department of Plant Virology, Institut Facultaire des Sciences Agronomiques, Kisangani-Yangambi, Kisangani, Democratic Republic of the Congo

**Keywords:** cassava, *Bemisia tabaci*, *Bemisia afer*, SNP, KASP

## Abstract

**Simple Summary:**

We report here the first detailed characterization of *Bemisia* whiteflies on cassava in eastern Democratic Republic of Congo (DRC). *Bemisia tabaci* transmits viruses responsible for devastating epidemics of cassava mosaic and brown streak diseases. KASP SNP genotyping of *B. tabaci* specimens collected from cassava in revealed the existence of four haplogroups, while phylogenetic analysis of mitochondrial *COI* sequences showed the presence of two major clusters of *B. afer*. This study provides important information on the genetic diversity of *B. tabaci* and *B. afer* in eastern DRC and emphasizes the need for continuous monitoring of whitefly populations on cassava to guide the strategic application of management practices that reduce the impact of cassava virus diseases throughout DRC and the wider Central Africa region.

**Abstract:**

The present study characterizes *Bemisia tabaci* and *Bemisia afer* from cassava in eastern Democratic Republic of Congo (DRC). The Mitochondrial *COI* sequencing revealed the occurrence of six cassava *B. tabaci* mitotypes, which were designated into four haplogroups (SSA-ECA, SSA-CA, SSA2, and SSA-ESA) using KASP SNP genotyping. SSA-ECA (72%) was the most prevalent and occurred in the northern part of the surveyed area, in the Ituri and Nord/Sud-Kivu provinces, whilst SSA-CA (21%) was present in the south, primarily in Haut-Katanga. SSA-ECA was predominant in the areas of north-eastern DRC most severely affected by cassava brown streak disease and was also reported in the new outbreak area in Pweto territory, Haut-Katanga, in the south. *Bemisia afer* comprised two major clusters with 85.5% of samples in cluster one, while the rest were in cluster two, which has no reference sequence in GenBank. This study provides important information on the genetic diversity of *B. tabaci* and *B. afer* in eastern DRC. This knowledge will be used as a basis for further studies to understand and to identify the role of whitefly haplogroups, their population densities and consequences for virus epidemics and spread as well as leading to improved vector and virus management strategies.

## 1. Introduction

Whiteflies (Hemiptera, Aleyrodidae) comprise more than 1500 species worldwide [1]. Some of these species cause significant crop damage directly through their feeding on phloem sap and are considered economically important pests [2,3,4]. In addition, whiteflies excrete honeydew, which leads to the proliferation of sooty mould fungi, discolouring and reducing the quality of food and fibre plants [2]. However, certain species, especially those within the *Bemisia tabaci* cryptic species complex, may cause major crop losses through the transmission of plant viruses [5,6] and are perhaps the most economically important pests of a wide range of crops, including cassava [2,7,8].

Cassava plants in Africa are colonized by several whitefly species, but the most predominant are *Bemisia tabaci* and *B. afer.* The greatest damage is caused by *B. tabaci* through the transmission of cassava mosaic begomoviruses (CMBs) [9,10] and cassava brown streak ipomoviruses (CBSIs) [11,12]. These viruses cause cassava mosaic disease (CMD) and cassava brown streak disease (CBSD), which together cause losses of over US$1 billion annually [13,14]. Super-abundant populations of *B. tabaci* not only are associated with severe virus epidemics in cassava but also cause physical damage through sap sucking and secretion of honeydew, which lead to the proliferation of sooty mould that impairs photosynthesis [15].

*Bemisia tabaci* comprises many morphologically similar populations but with distinct mitotypes (phylogenetic classification based on *COI* sequencing) that have been identified based on sequences of the mitochondrial cytochrome oxidase I (*COI*) gene [16,17]. Cassava-colonizing *B. tabaci* in Africa has been categorized, based on *COI* sequences, into five mitotypes, named sub-Saharan Africa 1 to 5 (SSA 1-5) [18,19,20]. SSA1 was further divided into five sub-groups: SSA1 sub-group1 (SSA1-SG1), SSA1-SG2, SSA1-SG3, SSA1-SG4 [20], and SSA1-SG5 [21]. SSA1-SG1 is the most predominant mitotype in most cassava-growing regions in East and Central Africa, including the Democratic Republic of Congo (DRC) and its neighbouring countries (Burundi, Central African Republic, Rwanda, Tanzania, and Uganda) [20,22] with the exception of South Sudan, which has SSA2 as the dominant mitotype on cassava [23]. Other mitotypes on cassava in these countries are SSA1-SG2, SSA2, SSA3, and SSA4 [18,20,22,23,24]. Recent studies [25,26] using SNP-genotyping through NextRAD sequencing identified six major genetic haplogroups (phylogenetic classification based on SNP-genotyping) and showed that *COI* is not effective at distinguishing the major genetic groupings of cassava *B. tabaci* in Africa. All the known mitotypes occurring on cassava were reassigned into the six SNP-based haplogroups: sub-Saharan Africa East and Central Africa (SSA-ECA), sub-Saharan Africa East and Southern Africa (SSA-ESA), sub-Saharan Africa Central Africa (SSA-CA), sub-Saharan Africa West Africa (SSA-WA), sub-Saharan Africa 2 (SSA2), and sub-Saharan Africa 4 (SSA4). A Kompetitive allele specific PCR assay (KASP) has been developed to distinguish the six major SNP-based haplogroups [27].

*Bemisia tabaci* is known to transmit over 300 economically important plant viruses, including begomoviruses, criniviruses, carlaviruses, torradoviruses, and ipomoviruses [6]. While virus transmission studies have confirmed *B. tabaci* as the vector of CBSIs, *B. afer* is not a vector of these viruses [28,29]. The CBSIs are transmitted in the field by whiteflies wherever CBSD occurs, and several *B. tabaci* genotypes occur across this geographical range [30], although studies on the transmission of CBSIs by different *B. tabaci* haplogroups have not been undertaken. The only comparison of the transmission of CMBs by *B. tabaci* whiteflies from different parts of Africa showed that there were no significant differences in transmission efficiency [29].

*Bemisia afer* is a species complex that is known worldwide and colonizes host plants that belong to over 22 families, including cassava [31,32]. Although it has been reported as a cassava pest that coexists with *B. tabaci* from various countries in Africa [33,34,35], it does not usually achieve sufficient levels of abundance to cause damage and has not been shown to transmit cassava viruses [35]. *B. afer* accounts for less than 20% of the whiteflies collected from cassava compared to *B. tabaci* [35]. However, it can be a possible source of error in linking whitefly abundance to virus disease spread because it can be readily misidentified in the field as *B. tabaci*. Based on *COI* sequencing, two *B. afer* genotypes have been reported on cassava in Africa, and these were distinct from those found in other parts of the world [32].

The eastern part of DRC is currently being affected by severe epidemics of CBSD that are expanding to previously unaffected regions [36,37]. Therefore, the objective of this study was to enhance knowledge of the whitefly vector in the epidemic expansion zones by characterising *B. tabaci* using mitochondrial *COI* sequencing and the newly developed KASP diagnostic [27]. This represents the first large-scale application of KASP to any insect pest in DRC.

## 2. Materials and Methods

### 2.1. Whitefly Sampling

Adults of cassava-colonizing whiteflies were collected from the shoot tips of 3–7-month-old cassava plants during surveys carried out in five provinces of DRC (Haut-Katanga, Ituri, Nord-Kivu, Sud-Kivu, and Tanganyika) from 2016 to 2018. Samples were collected from 106 cassava farmer fields at intervals of 5–10 km along motorable roads traversing each sampling area. At least 50 adult whiteflies (sucked using a pooter from the top five leaves of the tallest shoot) were collected per field from cassava plants randomly selected at nearly regular paces covering all sections of the visited field. The insects were immobilized in 95% ethanol and transferred into 1 mL screw cap vials before being transported to the laboratory at the International Institute of Tropical Agriculture (IITA)-Kalambo and stored in the freezer at −20 °C. The whiteflies were subsequently sent to IITA-Tanzania where they were processed for DNA extraction, PCR, *COI* sequencing, and KASP genotyping.

### 2.2. DNA Extraction

DNA was extracted separately from two female adult whiteflies that were selected from each vial, representing a single farmer’s field, making up to 212 insects selected in total. The insects were macerated in 20 µL of lysis buffer in a 1.5 mL Eppendorf tube. The lysis buffer contained 10 mM Tris-HCl (pH 8.0), 50 mM KCl, 2.5 mM MgCl_2_, 0.45% Tween-20, 0.01% Gelatine, and 60 µg/mL Proteinase K. The mixture was then vortex shaken, spun down, and immediately incubated on ice for 15 min. This was followed by incubation at 55 °C in a water bath for 30 min. The lysate was stored at −20 °C for downstream use. For PCR use, the lysate was diluted using sterile Diethyl pyrocarbonate (DPEC)-treated water in a ratio of 1:9. The samples from each province out of the 212 were Haut-Katanga (56), Ituri (42), Nord-Kivu (40), Sud-Kivu (50), and Tanganyika (24).

### 2.3. Mitochondrial Cytochrome Oxidase I (COI) PCR Amplification and Sequencing

A partial fragment of *COI* was amplified using one set of primers, 2195-Bt-F (5′-TGRTTTTTTGGTCATCCRGAAGT-3′) and C012-Bt-sh2-R (5′-TTTACTGCACTTTCTGCC-3′) [38]. These primers amplified a ~867 nt portion of the *COI* gene. The PCR reaction contained 1X QuickLoad Master Mix (New England Biolabs, Ipswich, MA, USA), 1 mM MgCl_2_, 0.24 µM of each primer, 2 µL DNA, and sterile distilled water to achieve the desired reaction volume of 25 µL. PCR was carried out at 95 °C for 5 min initial denaturation of template DNA, followed by 35 cycles at 94 °C for 40 s, 56 °C for 30 s for annealing, and 72 °C for 90 s for extension, with a final extension at 72 °C for 10 min. The PCR products were run on a 1% agarose gel in 1×TAE buffer stained with GelRed (Biotium, Fremont, CA, USA). DNA bands were visualized while using a Gel Doc XR+ Gel Documentation System. Out of the 212 samples, 200 generated clear bright bands of PCR products, which were sent to Macrogen Inc. (Rockville, MD, USA) for purification, and direct sequencing in forward and reverse directions. DNA sequences were manually edited using Ridom Trace Edit v1.1.0 (Ridom GmbH., Würzburg, Germany). One hundred eighty-three samples out of the two hundred had good sequences suitable for phylogenetic analysis. The sequences were assembled into contigs using CLC Main Workbench 7.0.2 (QIAGEN, Aarhus, Denmark). Multiple alignments of the edited sequences were performed using ClustalW in MEGA version 7.0.26 [39], and the sequences were trimmed to 708 nt. Construction of a maximum-likelihood phylogenetic tree was performed using MEGA with 1000 bootstrap replicates. Sequences were blasted using GenBank’s (NCBI) Blastn, and selected reference sequences with 99% to 100% identity to our *COI* sequences were included in the phylogenetic tree for comparison with previously published mitotypes. The number of *B. afer* haplotypes (phylogenetic classification of *COI* sequences with 100% nucleotide identity) was determined using Dna-SP 6.12.03 [40].

### 2.4. KASP SNP Genotyping of Cassava-Colonizing B. tabaci

The KASP assay currently uses six sites/SNPs to designate cassava-colonizing *B. tabaci* into the six haplogroups. This assay is only currently applicable to cassava-colonizing *B. tabaci* and not on other cryptic species or *B. afer*.

Whiteflies that were designated as cassava *B. tabaci* by *COI* sequencing were tested using KASP with a set of six primers (BTS99-319, BTS22-762, BTS141, BTS55-473, BTS613, and BTS46203), as described in Wosula et al. [27]. Conventional PCR primers were used to generate PCR products of genome portions containing target SNPs, and the PCR products were then used as DNA templates in KASP genotyping [27]. The KASP reaction mixture (10 µL) contained 5 µL of the 2X KASP master mix, 0.14 µL of the KASP primer assay mix, and 5 µL of the DNA template (1 µL of PCR product/DNA extract + 4 µL of sterile water).

KASP genotyping was performed in a Stratagene MX 3000P (Agilent Technologies, Santa Clara, CA, USA). The following cycling conditions were used: Stage 1: 30 °C 60 s (pre-read); Stage 2: 94 °C for 15 min hot-start Taq activation (1 cycle); Stage 3: 94 °C for 20 s, 61 °C (61 °C decreasing 0.6 °C per cycle to achieve a final annealing/extension temperature of 55 °C) for 60 s (10 cycles); Stage 4: 94 °C for 20 s, 55 °C for 60 s (29 cycles); Stage 5: 94 °C for 20 s, 57 °C for 60 s (3 cycles); and Stage 6: 37 °C for 60 s (1 cycle, cooling) followed by an end-point fluorescent read. These conditions were used for four primers (BTS99-319, BTS22-762, BTS55-473, BTS141), while Stage 3: 94 °C for 20 s, 68 °C (68 °C decreasing 0.6 °C per cycle to achieve a final annealing/extension temperature of 62 °C) was used for two primers: BTS613 and BTS46-203. The quality of genotyping cluster plots was visually assessed and only samples in distinct clusters with respective positive controls were considered for manual SNP calling using the MxPro software incorporated into the Stratagene MX 3000P unit.

Geo-referenced coordinates for samples successfully designated by KASP SNP genotyping were used to generate maps using ArcGIS 10.1 (ESRI, Redlands, CA, USA). Maps were produced illustrating the geographic distributions in eastern DRC of SNP-based haplogroups (KASP) data for cassava *B. tabaci* whiteflies. Geo-referenced co-ordinates for sites that were surveyed for whitefly abundance and CBSD incidence in 2016 and 2018 based on previously published data [37] were used to generate distribution maps for whitefly abundance and CBSD incidence in eastern DRC to complement the KASP SNP genotyping data.

## 3. Results

### 3.1. COI Mitotypes of Cassava-Colonizing B. tabaci in Eastern DRC

**Figure 1:**
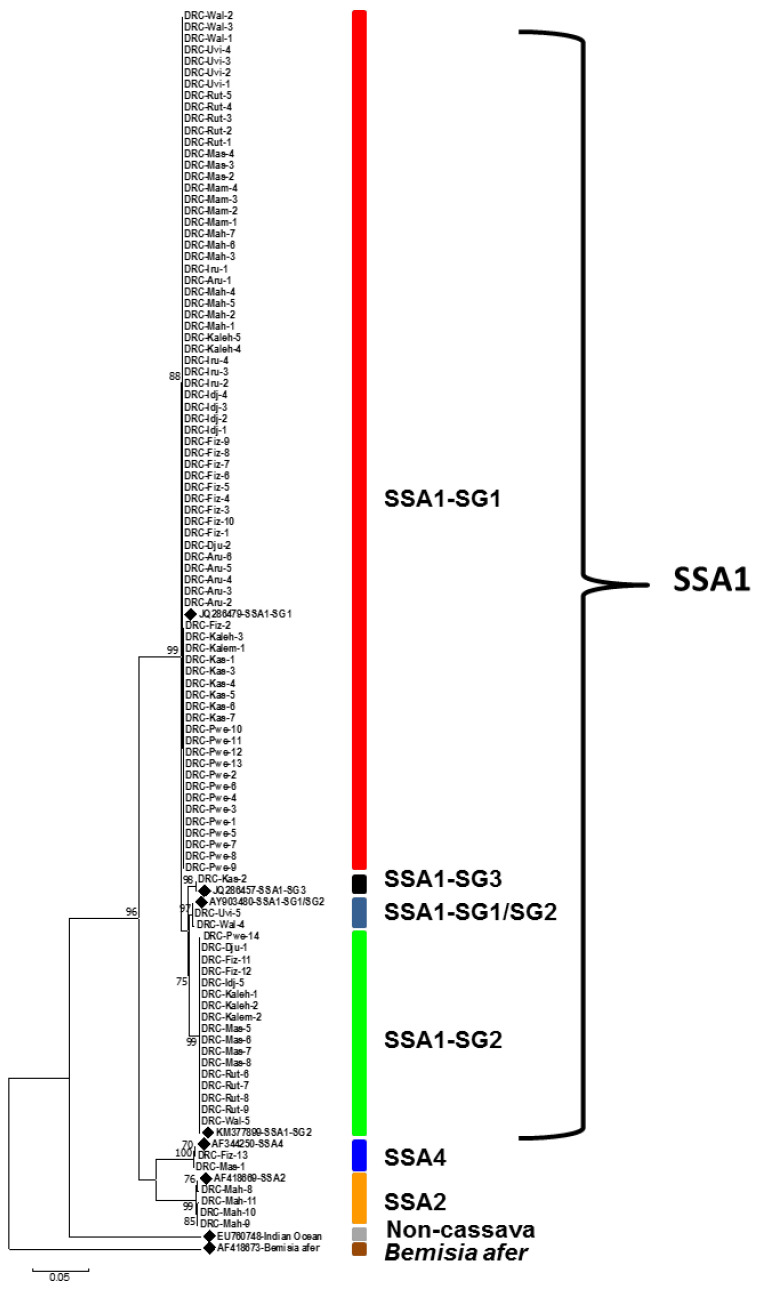
Maximum likelihood phylogenetic tree constructed for *COI* sequences obtained from *Bemisia tabaci* collected from eastern Congo between March 2016 and September 2018. Reference sequences from GenBank (♦) are included for comparison.

Sanger sequencing of *COI* for 200 whitefly samples generated 183 good sequences, out of which 100 were cassava *B. tabaci* (Haut-Katanga (21), Ituri (26), Nord-Kivu (16), Sud-Kivu (35), and Tanganyika (2)) and 83 were *B. afer tabaci* (Haut-Katanga (32), Ituri (10), Nord-Kivu (15), Sud-Kivu (13), and Tanganyika (13)). The phylogenetic tree of cassava *B. tabaci* showed three distinct mitotypes (SSA1, SSA2, and SSA4) of cassava *B. tabaci.* The major SSA1 mitotype had four sub-groups, which were SSA1-SG1 (74%), SSA1-SG1/SG2 (2%), SSA1-SG3 (1%), and SSA1-SG2 (17%). SSA2 accounted for 4% while SSA4 was 2% (Figure 1). SSA1-SG1 was found in all five provinces covered in the research area, with the Ituri and Sud-Kivu provinces accounting for 62%. SSA1-SG2 was found in four provinces (Ituri, Nord-Kivu, Sud-Kivu, and Haut-Katanga); SSA2 was recorded only in Ituri province; SSA4 was recorded in the Sud-Kivu and Nord-Kivu provinces; SSA1-SG1/SG2 was recorded only in Sud-Kivu province; and SSA1-SG3 was recorded only in Haut-Katanga province.

### 3.2. KASP Genotyping of Cassava-Colonizing B. tabaci in Eastern DRC

**Figure 2:**
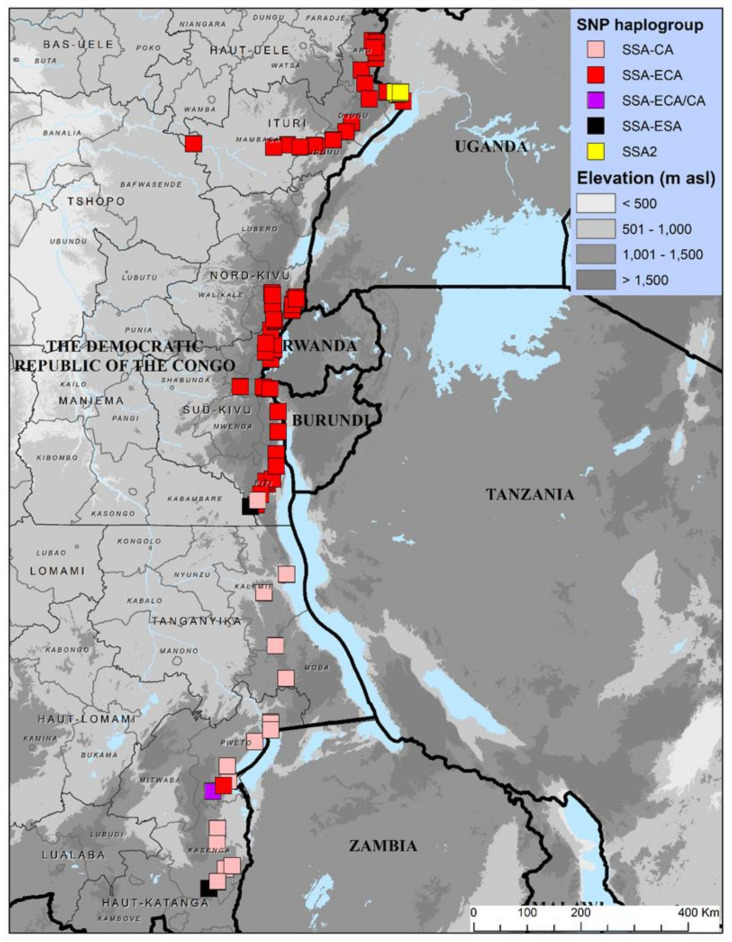
Geographic distribution of cassava-colonizing *Bemisia tabaci* whiteflies in provinces surveyed in 2016 and 2018 in eastern Congo, based on KASP SNP genotyping.

The 100 whiteflies identified as cassava *B. tabaci* by *COI* sequencing were designated into four haplogroups (SSA-ECA, SSA-CA, SSA-ESA, and SSA2) using KASP, except for a single sample that was not distinctly placed. SSA-ECA made up 72% of the samples. The SSA-ECA haplogroup (72 samples) mainly occurred in two provinces—Sud-Kivu (44.4%) and Ituri (31.9%)—while the remaining 22.2% were in Nord-Kivu and 1.4% in Haut-Katanga. SSA-CA comprised 21% of the 100 samples. The SSA-CA haplogroup (21%) occurred mainly in Haut-Katanga province (85.7%), while the remaining 14.3% were found in Tanganyika and Sud-Kivu. The haplogroup SSA-ESA had only two samples with one each in Sud-Kivu and Haut-Katanga provinces. There were four samples of SSA2, all in Ituri province. One sample from Haut-Katanga was not clearly distinguished. The SNP haplogroups map illustrates the predominance of SSA-ECA, which, apart from one sample, was restricted to the three northerly provinces of Ituri, Nord-Kivu, and Sud-Kivu, while SSA-CA was restricted to the two southerly provinces of Tanganyika and Haut-Katanga. Other SNP haplogroups with less than five samples each were SSA2 (Ituri), SSA-ESA (Sud-Kivu/Haut-Katanga), and SSA-ECA/CA (Haut-Katanga) (Figure 2).

**Figure 3:**
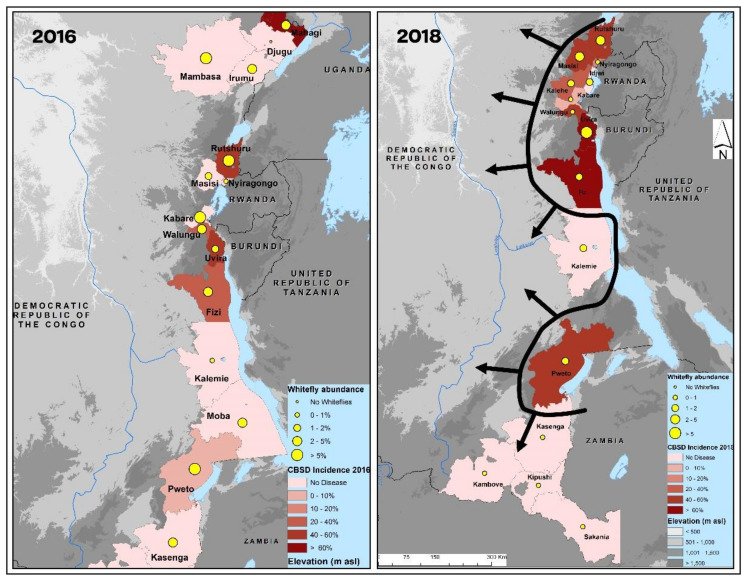
Maps illustrating the incidence of cassava brown streak disease (CBSD) and abundance of *B. tabaci* whiteflies on cassava in territories surveyed in 2016 and 2018 in eastern Congo.

Although determining the CBSI transmission characteristics of the haplogroups of cassava-colonizing *B. tabaci* was beyond the scope of this study, recently published data on CBSD epidemiology in eastern DRC demonstrate the greater abundance of *B. tabaci* in areas where CBSD incidence is higher (Figure 3; [37]). In general, there were more whiteflies in the northern parts of the surveyed region (Ituri, Nord-Kivu, and Sud-Kivu) and less in the south, while abundance in the south was greatest in Pweto territory (Haut-Katanga province), which is the most recent part of eastern DRC to be affected by CBSD. Whiteflies in the parts of eastern DRC with the greatest incidences are haplogroup SSA-ECA, while those in regions less affected by CBSD or unaffected are SSA-CA. The only exception to this geographical split was the presence of SSA-ECA in Pweto territory from the southern part of eastern DRC. It is significant that this is the area where CBSD is present and has increased greatly in incidence from 2016 to 2018.

### 3.3. Genetic Diversity of Cassava-Colonizing B. afer in Eastern DRC

**Figure 4:**
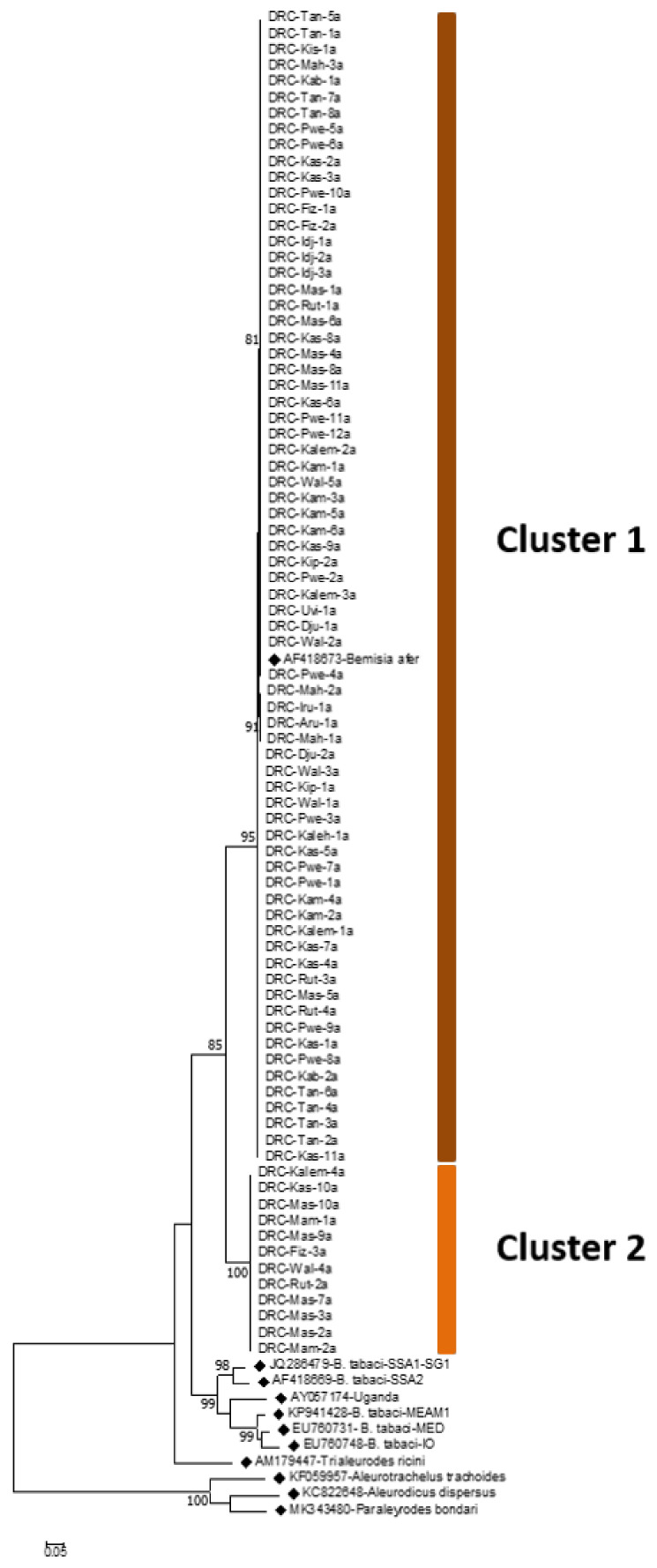
Maximum likelihood phylogenetic tree constructed for *COI* sequences obtained from *Bemisia afer* collected from eastern Congo between March 2016 and September 2018. Reference sequences from GenBank (♦) are included for comparison.

The phylogenetic tree of 83 samples shows two major clusters of *B. afer* (Figure 4). Cluster one with three sub-groups comprised 85.5% of the samples, with one sub-group having matching sequences in GenBank. Cluster 2 comprised 14.5% of the samples and had no matching reference sequence in GenBank, with the closest (AJ842052-602 nt) having 80% query cover and 87.3% identity (Figure 4). *B. afer* was most frequent in the southern province of Haut-Katanga, which accounted for 37.4% of the samples. The proportions for the other provinces were 12% for Ituri, 18% for Nord-Kivu, 15.7% for Sud-Kivu, and 16.9% for Tanganyika. The percentages of *B. afer* in each province in proportion to total whiteflies (*B. afer* plus *B. tabaci*) identified were 78% for Tanganyika, 54% for Haut-Katanga, 43% for Nord-Kivu, 24% for Sud-Kivu, and 27% for Ituri. 

A haplotype analysis of *B. after* *COI* sequences revealed the existence of five haplotypes, with one haplotype having a matching reference sequence, AF418673 from Uganda. This made up 49.4% of the samples. The other four haplotypes did not have any matching sequences in the database and can therefore be considered novel haplotypes. One of the haplotypes with 12 samples is a distinct cluster, with only 86.4% nucleotide similarity to samples in cluster one and the reference sequence AF418673. A total of 15 selected sequences that represent the five haplotypes of *B. afer* found in this study have been submitted to GenBank under the following accession names: MT985999-MT986013.

## 4. Discussion

This study reports, for the first time, a detailed picture of the genetic diversity of cassava-colonizing *Bemisia tabaci* in five provinces in eastern DRC. This is of significance since this is the part of Africa where there is the most active spread of CBSD, which is considered one of the world’s most dangerous crop diseases [41]. The three major mitotype groups based on *COI* sequencing (SSA1, SSA2, and SSA4) and the SSA1 subgroups (SSA1-SG1, SSA1-SG2, SSA1-SG1/SG2, and SSA1-SG3) reported in this study are consistent with what has been found for cassava-colonizing *B. tabaci* in other cassava growing regions/countries in sub-Saharan Africa [20,22,23,24].

The KASP genotyping of samples in this study further revealed the need to adopt this technique as a diagnostic assay for cassava *B. tabaci* across Africa [27]. The current study presents a more detailed picture of the respective distributions of SSA-ECA and SSA-CA than has been recorded in previous studies [25,26,27]. Perhaps most significantly, we have reported here the occurrence of SSA-ECA in individuals more than 500 km further south than the previous most southerly record of SSA-ECA [26]. Overall, the results suggest that the southern provinces of eastern DRC are a zone in which the ranges of distinct *B. tabaci* genotypes overlap.

*Bemisia tabaci* continues to be one of the most important agricultural pests in Africa as it transmits CMBs and CBSIs that cause major crop losses in cassava-production systems. Eastern DRC represents the ‘frontline’ in the westwards and southwards spread of CBSD [36,37]. There is a clear spatial association between the provinces most severely affected by CBSD (Ituri, Nord-Kivu, and Sud-Kivu) and the area of occurrence of SSA-ECA. Recent reports of CBSD have been made from both Haut-Katanga province [37] and the part of northern Zambia bordering Haut-Katanga [42]. Although SSA-CA was predominant in Haut-Katanga, it was notable that one individual case of SSA-ECA was also recorded there. Further testing of whiteflies from this border zone of south-eastern DRC and northern Zambia could help to clarify this apparent link between SSA-ECA and the CBSD outbreak in this region. An assessment of recently published data on CBSD epidemiology in eastern DRC also demonstrates the association between high whitefly abundance and CBSD incidence, particularly the link between whitefly abundance and CBSD incidence in the southern territory of Pweto [37]. However, future studies are required to determine the relative biological fitness and CBSI transmission efficiency of SSA-ECA and other major *B. tabaci* haplogroups in order to relate their occurrence with the risk of CBSD spread. However, earlier evidence has shown that cassava viral epidemics are associated with high abundances of whiteflies [14,43] and that transmission efficiency of haplogroups may not have a significant effect [29]. A study comparing the transmission of cassava mosaic begomoviruses (Africa cassava mosaic virus—ACMV and East Africa Cassava Mosaic Virus—EACMV) by three different mitotypes collected from Uganda, south Tanzania, and Ghana revealed that there were no significant differences [30].

This study reports, for the first time, the genetic diversity of *B. afer* whitefly populations in cassava-growing territories in eastern DRC. It reveals the existence of two major clades of *B. afer* on cassava in DRC. Two clades of *B. afer* were previously reported in samples from Malawi, Tanzania, and Uganda [32]. This is only the second study to characterise the genetic diversity of *B. afer* in Africa, where a greater focus has been placed on *B. tabaci* due to the magnitude of damage caused through the transmission of CMBs and CBSIs. Although there has been much less research attention on *B. afer* compared to *B. tabaci*, it is nevertheless important to have an improved understanding of this species, both since it can be readily confused with *B. tabaci* and since it is known to be an economically important viral vector in other cropping environments, transmitting the crinivirus, the sweet potato chlorotic stunt virus found in sweet potatoes in Peru [44]. Sequencing data from this study suggest that *B. afer* comprises approximately 45% of the whiteflies collected on cassava in eastern DRC and is predominant in the provinces of Tanganyika and Haut-Katanga compared to cassava *B. tabaci*. Changes in virus/vector affinities can occur as both evolve over time, and new viruses can be introduced to cropping systems where they did not previously occur. It is therefore important for future studies to monitor viral transmission by the identified species and haplogroups within those species. The findings from the study reported here contribute to improving knowledge of whitefly cryptic species in some of the most important cassava-growing regions of sub-Saharan Africa and provide vital baseline knowledge to help track current and future viral epidemics and support virus- and vector-management strategies to control those epidemics.

## 5. Conclusions

This study presents in detail the diversity of cassava-colonizing *B. tabaci* in eastern DRC. The *COI* sequencing, which has been used in most studies, designated these whiteflies into mitotypes previously reported in other countries, with SSA1-SG1 as the most predominant. KASP SNP-genotyping revealed the existence of four haplogroups of cassava-colonizing *B. tabaci* whiteflies with the two most abundant, SSA-ECA and SSA-CA, as the most predominant in the northern and the southern parts of the region, respectively. The northern region dominated by SSA-ECA is also the part of DRC most affected by spreading CBSD epidemics. This study also reports the detailed characterization of *B. afer* on cassava, another common whitefly species that is largely ignored. However, knowledge of its dynamics, genetic characteristics, and distribution are important for researchers conducting research on cassava whiteflies, which collectively are some of the most economically important insect species on the African continent. The findings from this study significantly expand the knowledge of cassava-colonizing whiteflies and their relationship with ongoing viral epidemics and contribute important baseline information for the development of virus vector management strategies.

## Data Availability

Partial *COI* Sequences representing five haplotypes of *Bemisia afer* obtained in this study were deposited in GenBank through online submission portal under accession numbers (MT985999-MT986013).
